# Community‐based adaptation research in the Canadian Arctic

**DOI:** 10.1002/wcc.376

**Published:** 2015-11-25

**Authors:** James D. Ford, Ellie Stephenson, Ashlee Cunsolo Willox, Victoria Edge, Khosrow Farahbakhsh, Christopher Furgal, Sherilee Harper, Susan Chatwood, Ian Mauro, Tristan Pearce, Stephanie Austin, Anna Bunce, Alejandra Bussalleu, Jahir Diaz, Kaitlyn Finner, Allan Gordon, Catherine Huet, Knut Kitching, Marie‐Pierre Lardeau, Graham McDowell, Ellen McDonald, Lesya Nakoneczny, Mya Sherman

**Affiliations:** ^1^ Department of GeographyMcGill UniversityMontrealCanada; ^2^ Departments of Nursing and Indigenous StudiesCape Breton UniversitySydneyCanada; ^3^ Department of Population MedicineUniversity of GuelphGuelphCanada; ^4^ Department of EngineeringUniversity of GuelphGuelphCanada; ^5^ Departments of Indigenous Studies & Environmental StudiesTrent UniversityPeterboroughCanada; ^6^ Institute for Circumpolar Health ResearchYellowknifeCanada; ^7^ Department of GeographyUniversity of WinnipegWinnipegCanada; ^8^ Sustainability Research CentreUniversity of the Sunshine CoastSippy DownsAustralia; ^9^ Faculty of Public HealthCayatano Heredia UniversityLimaPeru

## Abstract

Community‐based adaptation (CBA) has emerged over the last decade as an approach to empowering communities to plan for and cope with the impacts of climate change. While such approaches have been widely advocated, few have critically examined the tensions and challenges that CBA brings. Responding to this gap, this article critically examines the use of CBA approaches with Inuit communities in Canada. We suggest that CBA holds significant promise to make adaptation research more democratic and responsive to local needs, providing a basis for developing locally appropriate adaptations based on local/indigenous and Western knowledge. Yet, we argue that CBA is not a panacea, and its common portrayal as such obscures its limitations, nuances, and challenges. Indeed, if uncritically adopted, CBA can potentially lead to maladaptation, may be inappropriate in some instances, can legitimize outside intervention and control, and may further marginalize communities. We identify responsibilities for researchers engaging in CBA work to manage these challenges, emphasizing the centrality of how knowledge is generated, the need for project flexibility and openness to change, and the importance of ensuring partnerships between researchers and communities are transparent. Researchers also need to be realistic about what CBA can achieve, and should not assume that research has a positive role to play in community adaptation just because it utilizes participatory approaches. *WIREs Clim Change* 2016, 7:175–191. doi: 10.1002/wcc.376

For further resources related to this article, please visit the WIREs website.

## INTRODUCTION

Climate change adaptation research seeks to identify and evaluate policies, measures, and strategies to reduce vulnerability and enhance adaptive capacity to climate change impacts.^1^ The importance of engaging communities and decision makers in this work is now widely recognized.^2^, ^3^, ^4^ On normative grounds, this is part of good governance, and on instrumental grounds, it is believed to be necessary for effective adaptation policy implementation and programming.^5^, ^6^ Indeed, adaptation has been described as a ‘wicked problem,’ involving complex interactions among societal and natural factors, where facts are uncertain, values in dispute, and decisions urgent.^7^ In this context, there are rarely single adaptation solutions; rather, adaptation involves multiple trade‐offs and spans multiple institutions and scales, with diverse perspectives on what constitutes ‘good’ adaptation.^7^, ^8^, ^9^ Central to addressing such problems is the production of knowledge in the context of its application, taking into account different forms of understanding, building upon knowledge of local conditions and decision‐making processes, and involving multiple disciplines and a variety of stakeholders.^10^, ^11^, ^12^ This process is essential for creating ‘usable science’ or ‘practice‐orientated research’ that is explicitly designed to inform adaptation decision making and policy, and to identify and support effective adaptation strategies.^13^, ^14^, ^15^, ^16^

The development of community‐based adaptation (CBA) approaches is one response to the call for more usable science for adaptation. Emerging from the participatory development field, and supported more broadly by calls for deliberative governance practices in society,^17^, ^18^, ^19^ CBA can be defined as ‘a community‐led process, based on communities’ priorities, needs, knowledge, and capacities, which should empower people to plan for and cope with the impacts of climate change.’^20^ Research has an important role in this process, where ‘research’ refers to systematic investigation or inquiry aimed at contributing to knowledge on climate change impacts, vulnerability, and adaptation, and which builds upon local, traditional, and scientific knowledge systems. Capacity building, knowledge mobilization, empowerment, and training are also viewed as essential features of research in CBA, with various actors performing research functions in CBA projects, including academics, consultants, nongovernmental organizations (NGOs), governments, and communities themselves. Implicit to CBA is some form of external engagement to assist communities in this process, and may range from projects where communities themselves request support to those that are initiated by outside actors. This reflects the nature of climate change, where despite communities often having significant knowledge on local environmental risks, understanding of projected climate change, and potential vulnerabilities is often limited.^21^

Reflecting the breadth of responses that adaptation to climate change can take at a local level, CBA projects take a variety of forms.^22^, ^23^, ^24^, ^25^ On the one hand, they may initiate discussion on the risks posed by climate change, raise awareness on the need to adapt to future stressors, build general capacity to identify and manage change, and seek to inform decisions undertaken at the individual, household, and community level. On the other hand, CBA projects may be deliberately supported by governments or donors to identify, prioritize, implement, and evaluate adaptations. Local level engagement and emphasis on local empowerment underpin CBA projects and, increasingly, effort is being directed to scaling‐up community work to inform broader level adaptation programing.^23^, ^26^ While most CBA projects have been undertaken in low‐to‐middle income nations, there is also emerging interest in employing CBA in high‐income nations, specifically with indigenous populations and resource‐dependent communities.^27^

The CBA literature promises much and, as Dodman and Mitlin^28^ note, its advocates argue that it is a highly effective approach for assisting adaptation and building adaptive capacity at a local level. As CBA has become mainstream, however, some have begun to question the *de facto* assumption that engaging communities in this manner is always the best or most successful approach for adaptation.^6^, ^22^, ^28^, ^29^, ^30^, ^31^, ^32^ Such reflections have been primarily articulated in the context of research in low‐to‐middle income nations, and critique the often uncritical way CBA is portrayed within research and practitioner communities, with limited examination of the power relationships that influence the outcomes of local level projects or analysis of the challenges associated with undertaking CBA in cross‐cultural contexts.^33^, ^34^, ^35^ There remains a need for further such reflective attention and to extend this to work taking place to indigenous populations in high‐income nations—what Young^36^ called the ‘third world in the first’ and others ‘the fourth world’^37^—to ensure that CBA is undertaken appropriately and productively with, by, and for indigenous peoples and communities. In the Arctic, e.g., community‐based research is being widely promoted by communities, governments, indigenous groups, and funding organizations; yet concerns have been noted over potential for tokenistic engagement of communities, consultation fatigue, and imbalance between Western and indigenous knowledge in this work.^13^, ^15^, ^38^, ^39^, ^40^

In this article, we respond to this identified deficit, critically examining the opportunities, tensions, and challenges that arise within CBA work with Inuit communities in Canada. The article aims to go beyond simply documenting successes and challenges to critically reflect and deliberate on aspects of CBA research from our collective experiences as Western‐trained nonindigenous academics who work within the Canadian Arctic and sub‐Arctic, as well as with indigenous partners globally. Such critical reflection is essential if we are to learn from our experiences, and provides a foundation for future projects that seek to work at the interface of Western and indigenous knowledge in an adaptation context in general and in the Arctic in particular. As such, the work is part of what Preston et al.^34^ call a reflexive approach to adaptation research; a nascent field of study in the adaptation field that targets ‘the research paradigm itself and the role of the researcher within it.’ We begin by providing context on Canada's Inuit populations and the climate adaptation challenges faced, then describe the multiday workshop methodology that provides the research and reflexive basis for the paper, and end with a critical examination of Inuit‐focused CBA research.

## CANADA'S INUIT POPULATION

Canada's Inuit population numbers approximately 59,000 people, primarily living in small, remote coastal communities scattered across Canada's northern coastline. Communities range in size from approximately 7000 to as low as 100, with the four Inuit‐administered settlement regions (Nunavut, Nunavik, Nunatsiavut, and the Inuvialuit Settlement Region) covering approximately 31% of the Canadian landmass. Many Inuit communities rely on mixed economies, composed of waged employment and subsistence activities.

### Inuit and Climate Change

Projections indicate that the Arctic will see the most rapid and extreme warming this century, at least double the global average, and this warming is expected to have substantial impacts on sea ice, extreme weather events, and Arctic ecology.^41^, ^42^ Already, Northern Canada—referred to also as ‘the North’—is experiencing some of the most dramatic changes in climate globally, with impacts on personal safety, food and water security, mental health and well‐being, and community infrastructure documented.^41^, ^43^, ^44^, ^45^, ^46^, ^47^, ^48^, ^49^ Socioeconomic conditions and change increase the sensitivity of Inuit communities to these impacts.^42^, ^48^, ^50^, ^51^ For example, research has documented high rates of food insecurity; housing overcrowding and poverty are chronic problems; wide‐ranging institutional challenges have been identified as constraints to community planning and health care provision; and access to health‐sustaining resources has been identified as a major challenge.^52^, ^53^, ^54^, ^55^, ^56^, ^57^ These challenges reflect, in part, the ongoing legacies of colonization as Inuit were settled into permanent communities and incorporated into a colonial relationship with the Canadian state in the 1950s and 1960s, and livelihoods and societal interactions transformed over a short period of time.^58^, ^59^

Despite well‐documented vulnerabilities to a changing climate across the North, research has also highlighted the substantial adaptability of Inuit communities.^42^ Indigenous knowledge and social networks, have been identified as protective factors, moderating sensitivity to climate‐related risks and underpinning adaptive capacity, albeit with concerns in‐light of continuing and often‐rapid socioeconomic and cultural transformations.^48^, ^60^, ^61^, ^62^, ^63^, ^64^, ^65^ Indeed, Northern societies have not been passive in the face of a rapidly changing climate, and adaptation has been prioritized as an essential component of Northern climate policy since the early 2000s, with the creation of adaptation plans for many Northern communities and a variety of adaptation policies, programmes, and practices evident.^66^, ^67^, ^68^ More broadly, Inuit have secured growing political autonomy, providing new forums to respond to Northern concerns and priorities, and have been vocal in lobbying for climate action on the national and international stage.^69^

### CBA in a Canadian Inuit Context

Significant research on the human dimensions of climate change has been undertaken in Northern Canada, and there is an emerging focus on adaptation policy development across different levels of government.^70^, ^71^ The importance of engaging communities throughout all stages of the research process, from setting and defining project priorities, to overseeing research activities, to collecting and analyzing data, to disseminating results, is increasingly being recognized, with the majority of projects claiming to adhere to principles of participatory research.^16^, ^39^, ^47^

In particular, the last decade has witnessed an increased emphasis on CBA research in Northern Canada, and is increasingly being emphasized by funding agencies, governments, research licensing bodies, and the scientific community. At a broad level, this builds from acknowledgment of the damage that historical research practices have caused in the North, of an understanding of continuing colonial legacies, and of associated mistrust of Western knowledge.^72^, ^73^, ^74^, ^75^, ^76^ Specific to adaptation, the importance of community engagement reflects how climate change will affect Northern communities, with many risks propagated through subsistence‐based hunting and land use activities in which individual, household, and community behavior and decision making are essential for adaptation to current and future change.^44^, ^46^, ^50^, ^66^, ^77^ Many adaptations, therefore, need to be rooted in local customs, values, and decision‐making process if they are to be successful, building on traditional knowledge of local environmental conditions and coping mechanisms. Local engagement is further underscored by institutional processes in Canada's Inuit regions, in which community consultation, the integration of cultural values, and respect for traditional knowledge are built into political and governmental decision‐making processes as part of land claims agreements and regional and territorial government institutions. In this context, adaptation policy and planning processes have significant emphasis on community engagement.^66^

CBA projects have taken a variety of forms in Inuit regions, including projects assessing vulnerability where the aim is to work with communities to illustrate how individual and household behavior, decision making, and social networks can underpin efforts to manage climate change impacts; projects actually developing, implementing, and evaluating adaptations supported by government programs; and initiatives focusing on building skills, fostering social learning for adaptation, and sharing knowledge on current and projected climate change impacts. A diversity of community–researcher partnership has evolved in CBA projects, with some guided by formally negotiated partnership agreements, although the majority of projects proceed based on informal agreements.

## METHODOLOGY

To provide the basis for critically examining CBA research with Inuit communities in northern Canada, a 2‐day workshop that brought together academic researchers and emerging scholars was held at McGill University. Participants (*n* = 23) were selected via their affiliation with a community‐based partnership project that combines Western and indigenous knowledge to inform adaptation policy and programming in the Canadian North, and included faculty members of varying years of experience from six Canadian universities and one Australian university (*n* = 8), masters and doctoral students (*n* = 8), graduate research assistants (*n* = 5), an academic based at a Northern research institution (*n* = 1), and one researcher in the federal government (*n* = 1). Students were included due to their often‐considerable fieldwork responsibilities in adaptation projects and their day‐to‐day engagement with communities. Attendees represented a variety of academic disciplines, including geography, epidemiology, population medicine, indigenous studies, engineering, and public health; have substantial experience working in the North and beyond on community‐based research, some for over 20 years and all have been working with Arctic communities for at least 1 year; have studied various risks posed by climate change; and, collectively, have worked with Inuit communities in all four Inuit regions across Canada. As such, the reflections of participants in the workshop are based upon a diversity of personal experiences gained from many projects in many different communities.

A workshop was selected as the most appropriate venue for critical reflection on CBA because it allowed multiple perspectives from diverse academics to be brought to the table and discussed in a collegial setting. In so doing, the work is consistent with other studies in the peer‐reviewed scholarship that have used workshops for knowledge generation and reflection (e.g., Refs 78, 79, 80). The inclusion of only academics reflected our aim to provide a space for researchers to reflect on the challenges faced in mobilizing CBA principles in their own work. The article, therefore, provides only a partial reflection of CBA, capturing the perspectives of university‐affiliated and government‐based nonindigenous scientists. We do not claim to speak for communities about their experiences in research, and recognize the importance of supporting Northern community members’ reflections on, and critiques about, the research process.

Facilitation of the workshop sought to promote a collaborative learning process with emphasis on reflection among participants, and encouraged a balance between structured discussion and exploration of emergent themes. The aim was to challenge and deliberate the role community‐based research can and should play in adaptation. A number of steps were used to this end. First, prior to the workshop, participants were asked to read four key articles^22^, ^39^, ^72^, ^81^ and were provided with the workshop format, as well as a list of questions to consider in advance. The workshop began with participants describing the projects they were engaged in, before they were divided into break‐out groups, preselected to have a diversity of members of different ages, years of experience, disciplines, and geographic focus of work. Each group was assigned a facilitator, and questions were posed to stimulate participants to share their experiences and to reflect on CBA as a group, with discretion given to each group to allow discussions to evolve according to the interests of those present. The breakout groups then reconvened, with each profiling the main thoughts and ideas they discussed, which were used for a facilitated full group discussion on key themes.

Detailed notes on the workshop were kept by all facilitators, and were synthesized and analyzed to identify key themes. Four meta‐themes were identified around which key arguments made in the workshop were profiled. Building on the emergent themes from the workshop, a literature review was then conducted, focusing on the general CBA literature and scholarship on participatory research approaches in indigenous communities, and was used as a basis for critically examining CBA work with Inuit communities.

## CRITICAL REFLECTIONS ON CBA

### CBA Builds upon a History of Community‐Based Participatory Research

It is important to note that CBA has not evolved in a vacuum in Northern Canada but, rather, builds upon work on community‐based participatory research (CBPR), where considerable scholarship on resource management, health, and community development has been conducted with indigenous communities, with substantial focus on how to ethically engage communities and work with indigenous knowledge, epistemologies, and cultural values.^82^ The focus on intervention, policy development, and planning for future risks—within a context of climate change impacts where uncertainty is high and recognition of change is sometimes contested—differentiates CBA from CBPR, and also brings additional challenges. Indeed, in the workshop, participants expressed unease at how CBA/CBPR terms were being appropriated by the research community (including, at times, by themselves), with many feeling they had become buzzwords that were often used tokenistically to sell projects to funders, with community engagement being viewed as a box to tick as opposed to an ongoing process of dialogue and engagement. Such concerns parallel discussions around the use of the terminology of ‘participation’ and ‘empowerment’ in the international development field or in adaptation studies more generally,^34^, ^83^, ^84^ and many felt that any academic with an adaptation focus was now obligated to frame their scholarship with a participatory research lens to meet these expectations. This raises problems, particularly if projects framed as participatory are not in practice, potentially leading to spurious findings and inappropriate recommendations.^34^

### The Role of Research in CBA

It is often taken as a truism that research has a positive role to play in community adaptation, yet there have been very few attempts to investigate many of the claims made.^4^, ^6^, ^28^ There is a long history of interventions in Northern Canada, often advanced by outsiders in the name of ‘beneficial’ outcomes, which only serve to reflect nonindigenous worldviews and notions of progress and planning. These outside interventions have led to critiques about how researchers approach ‘community’ in the context of climate change and other issues.^38^, ^85^, ^86^, ^87^ In the workshop, this emerged as central theme, with participants reporting concern about how adaptation discourse has the potential to perpetuate legitimization of outside intervention/control, and entrench unequal power relations between Northerners and outside researchers. Many reported a general feeling of unease at being involved in research intervening in communities and cultures of which they are not a part, particularly about appropriating local initiatives as research projects and potentially compromising community leadership and agency.

The effectiveness of research in enhancing adaptive capacity can be problematic given the considerable socioeconomic barriers to adaptation at various scales and over which communities and researchers have limited influence.^22^, ^84^ Participants also expressed concern that CBA could establish adaptation as a local issue, and depoliticize structural determinants of adaptive capacity. This led some in the workshop to question the ethics of the adaptation/intervention framing of CBA projects, given the difficulty of achieving positive change in the short term or within the confines of funding cycles, and possibility of ‘over‐promising’ results or outcomes from projects.

The sustainability of projects is also a concern for CBA. A number of workshop participants reported working with communities on research‐funded projects to implement and evaluate local pilot adaptation initiatives. In one example, the pilot program was widely acclaimed by the community and showed demonstrable links to enhancing adaptive capacity. While the aim of the work was to showcase and demonstrate the effectiveness of the initiative as a potential adaptation, upon project completion there was widespread expectation the initiative would continue. Despite repeated attempts, additional funds or a government partner to continue the work could not be found. Without funding to support needed supplies and materials, and community member employment, the project dissolved, resulting in feelings of loss and lack of power among the participants engaged and the communities in which the pilot strategy was implemented, potentially undermining long‐term adaptive capacity.

The preceding example raises questions about the role of research in adaptation in absence of broader level of support and commitment from various levels of government; a context within which much CBA work takes place, even in the North where there is strong emphasis on responding to community needs at various levels of government. CBA faces a dilemma here: research is needed to develop an evidence base on adaptation, yet the very participatory process used to create this evidence base can generate significant expectations for positive change, which, if not followed with visible developments, can reduce local interest in and valuation of research or, worse, become maladaptive (Table 1). Moreover, with research having an increasingly active role in developing and funding interventions, there is the potential for decision makers at various levels of government to download adaptation to the research community, a role researchers neither have the mandate, capacity, or legitimate authority to take on. There has been significant debate in the general literature about the ways in which the local focus of CBA can direct attention away from underlying structural determinants of vulnerability.^84^, ^88^, ^89^ The substantial research focus on developing community adaptation may also foreclose opportunity for local institutions to spearhead this work independently, perpetuating colonial patronage relationships and uneven power dynamics between Northern and academic research partners.

**Table 1:** Potential Pathways through Which CBA Can Lead to Maladaptation

Challenge	Potential Pathway to Maladaptation
Community engagement seen as an obligation because of historical colonial research practices that created mistrust between researchers and locals	Adaptation depoliticized from its broader structural determinants (colonization, poverty, and inequality)
Licensing processes often require researchers to engage in collaborative research, thereby institutionalizing CBA	Many academics may not be trained or committed to CBA, which may facilitate tokenistic interaction with communities, ‘consultation fatigue’ and conflict with local values of meaningful reciprocity
Adaptation is downloaded from broader levels of government to researchers and communities	Adaptation established as a local issue leaving the barriers to local action at regional to national levels unaddressed
Researchers and communities lack funds and long‐term time frame to support adaptation such that intervention do not materialize or have short duration	Community interest in research drops; sense of loss on project completion emphasizes lack of power at community level
Emphasis in the literature on successful projects does not provide full disclosure of complexity of CBA research and practice	Lack of reporting on challenges and failures in CBA and associated ‘lessons learned’ to help refine future research design and implementation
Adaptation focus and integration of future concerns diluted in response to different community interests	Policies developed which do not adequately address projected future changes; pertinent climate change risks overlooked; adaptation research focus compromised
Overprivileging of Western knowledge if power relations unaddressed, and it is assumed the participation on its own will lead to good adaptation	Undermining of determinants of adaptive capacity including cultural norms and traditional knowledge; lack of community ownership of proposed adaptations; decreased trust in Western knowledge
Intervention‐orientated focus of CBA can reduce space for local leadership	Perpetuation of uneven power dynamics between Northern and academic research partners

The role of research in community adaptation is thus complex, and some have questioned whether communities would be better off without this type of research. Consensus emerged in the workshop that CBA *does* have a role, but not in all cases and is contingent upon community experience with past research, the nature of the proposed work, and process of engagement, community interest, and priorities. Deciding when and how to intervene in adaptation processes through research should be carefully considered with an eye toward a number of the benefits and harms that may arise. Such reflection needs to be an explicit goal in the early stages of proposed CBA work.

Specific benefits research can bring within the context of Inuit‐focused CBA projects include:

- *Practical information and benefits*: There are a number of practical reasons for researchers being involved in community adaptation, including the ability to facilitate financial and human resources to which communities otherwise would not have access; the focus on issues which would likely be neglected given the present day nonclimate‐related challenges facing Northern settlements; providing support to interpret jargon‐laden scientific reporting about climate change into meaningful information; the ability to advocate for policy needs to broader levels of government; the opportunity to link communities in different regions facing similar challenges with climate change; the ability for researchers to be more neutral and work across kinship boundaries in communities; and the potential to help define and pursue answers to research questions of relevance to community members, especially where incomplete or inaccurate information constrains adaptation. Furthermore, given historical experiences of research in the North, the focus on working with communities in projects in which indigenous knowledge is highly valued can help to legitimize and enhance confidence in such knowledge systems to the science community in general, to policy makers, and in some instances to communities themselves (e.g., Ref 81).
- *Future focus*: While environmental change in itself is not new in the North, and while indigenous knowledge and practice have evolved and continue to evolve in this context,^90^ the magnitude of projected changes in climate and their long‐term directional nature may challenge Inuit conceptualizations and understanding of environmental (in)stability and government planning.^91^, ^92^ Research can have an essential role in facilitating consideration of how projected future changes may impact communities and livelihoods, affect government policy and programming, and challenge the rights of Inuit (e.g., as per land claims agreements).
- *Evaluation*: The development of an evidence base on the potential effectiveness of community adaptations based on the integration of Western and indigenous knowledge is an important area within which research can contribute. This is particularly important if higher levels of government are to support adaptation programming in the context of other pressing needs.
- *Accountability across scales*: CBA prioritizes the community as the entry point for adaptation, yet responses in one location can displace impacts and vulnerabilities to other communities, regions, or to future generations.^92^, ^93^ Research has an important role and a responsibility in examining the extent to which community adaptations are optimal across sectors and regions, and within the context of current and projected stressors, and of being mindful of how potential adaptation recommendations or strategies could impact other areas.

### The Challenges and Limitations of CBA Research in the North

That CBA can have a positive role in community adaptation is widely acknowledged in the literature and by participants in the workshop, but it is important to note that CBA is not a panacea as sometime presented in the literature, and has limitations and potential negative impacts. Indeed, participants in the workshop believed that the often uncritical belief that CBA approaches are ‘better,’ is reducing space for critical reflection on which approaches and methods are most appropriate for a given context and question. For instance, not all communities want or have the capacity to be involved to the level of engagement implied by CBA approaches, or are they necessarily suitable or desirable for all research questions. Furthermore, participation also engenders challenges: personal views may become more entrenched through participation if not structured properly, participants may become more confused through exposure to different viewpoints and ideas, and local power dynamics (which may go unnoticed or unappreciated by outsiders) may preclude the inclusion of all voices in the process or engagement maybe limited to most politically active and interested community members.^31^, ^35^, ^94^ Arguably, in some cases, more conventional researcher‐led projects may have a more important role to play in adaptation research, especially where communities actually want someone to independently respond to a pressing issue or need; yet in the context of the current literature, this might be deemed ‘inappropriate.’ Similar concerns have been recently articulated in the literature with regards to practice‐orientated approaches to adaptation in general.^4^, ^31^, ^34^

It is also important to note that researchers should not expect everyone in communities to welcome the proposed work or be interested in engaging, however participatory the process attempts to be. With regards to this latter point, some student participants noted significant anxiety during their fieldwork—despite following guidelines for community engagement—as local interest in being engaged was limited; indeed, the literature, with its overemphasis on success stories and sanitized descriptions of methodology, had not prepared many students for the on‐the‐ground realities of community research. Beyond practical challenges of doing participatory research in the North—time commitment, expense, language differences, and institutional recognition and support (e.g., see Refs 34, 39, 40, 95, 96)—the nature of CBA can also bring tensions to researcher‐community relations, including:

- *Tensions over process*: Given the in‐depth nature of community engagement in CBA, there is the potential for community resistance to certain components of research. Workshop participants, e.g., described how communities viewed evaluation of pilot adaptations as intrusive and distracting despite recognition by local leaders and institutions of the importance of such metrics. Accommodating these concerns often involves scaling‐down the depth and frequency of the evaluation process and tools (e.g., fewer interviews, no large community surveys), which may potentially compromise the ability to generate results of sufficient rigor to assist communities in their efforts to obtain funding to continue pilot interventions or to scale up pilot projects into regular programs. There is also a tension between recognizing the importance of considering future risks, while still understanding that there are more pressing day‐to‐day issues that may take precedence. This is further complicated by the fact that, for some Inuit, there is a strong hesitancy to speak about the future due to a belief in the sentience of the natural world, acknowledgment of variation and the transient nature of environmental knowledge, and cultural resistance to focusing on the negative.^38^ Consequently, few of the adaptation projects that workshop participants were engaged in had a strong future focus, despite this being recognized as a key contribution researchers can bring to community adaptation. A similar observation has been made in the general CBA literature where the factoring of future climate risks into CBA has remained limited, and even discouraged by some for fears that such consideration may lead to overemphasis on future‐orientated technological responses.^32^, ^97^, ^98^
- *Maintaining a climate change*/*adaptation focus*: Despite the fact that the Arctic is witnessing the most dramatic climate change globally, communities are not always interested in climate change‐focused projects, with more pressing issues often requiring attention.^99^ Consequently, some workshop participants felt like they were almost pushing adaptation onto communities in their work. There was acknowledgement that maintaining an adaptation focus can be challenging given the participatory nature of CBA projects, in which communities can steer attention to other issues that may be more pressing at the current time (e.g., Ref 100).

The tensions of having adaptation as the entry point for projects raise questions over whether climate change is actually a contrived framing. Indeed, there is an absence of discussion in the literature on how an adaptation framing is developed and maintained in projects—where it is typically assumed that communities *are* interested and *are* willing to play a major role in leading adaptation projects. Many workshop participants, based on their own experiences, doubted this was always the case, believing the lack of critical discussion to reflect the common assumption that community‐initiated and ‐led projects are ‘better,’ and without which good participatory research cannot occur. Locally initiated and led projects are certainly an important dimension of CBA, but close and effective participation may also occur where researchers themselves initiate an adaptation project, providing engagement is done in a collaborative and ethically sound manner. Particularly for creeping and slow‐onset hazards like climate change, where there are weak incentives for institutions and individuals to mobilize given the uncertain long‐term nature of the problem, some outside initiation is often essential.^5^, ^32^, ^101^, ^102^

- *Conflicting results*: CBA often involves knowledge being created at the interface of Western and local/indigenous knowledge within a specific cultural context.^103^ There is significant potential for both knowledge systems to be complementary when understanding the nature of the risks posed by climate change or identifying adaptation strategies (e.g., see Refs 104, 105, 106). In some areas, however, there remains divergent and polarized opinions on current and projected impacts of climate change.^107^, ^108^, ^109^ In these cases, the need to adapt is contested, and preferred short‐term policy responses may compromise long‐term sustainability. The role of research in such situations is complex.^32^, ^103^ Building upon and integrating indigenous and Western knowledge underpin CBA, but researchers, communities, and decision makers may have different perspectives on risks, particularly once climate projections are integrated, reflecting different value systems about the future. In such circumstances, communities may not want to hear results that could imply the need for significant changes in livelihood activities, or may request the results be kept confidential; equally, researchers may dispute findings if they are not corroborated by other research. Such circumstances are not unique to the North; Few et al.^110^, ^111^ document similar challenges in the context of coastal management in the UK where communities often want to ‘wait and see’ as opposed to getting involved in anticipatory adaptation action. These challenges require careful and skilled negotiation, especially given the policy implications of research, and in an Arctic context, the uncertainty about future wildlife trends. Indeed, communities often highlight the inaccuracy of past scientific stock assessments, which underpin the mistrust many Inuit feel for conventional scientific research.^112^, ^113^, ^114^

One implication concerning tension over results is that CBA projects may avoid the more contentious issues or try to minimize ‘rocking the boat.’ The potential for such tension should not, however, distract from the benefits of CBA; rather, the example of wildlife management further emphasizes the importance of the *process* of CBA involving continual dialogue and transparency between researchers and communities, beginning at project inception, so that all parties are aware of the direction the work may take and offer their free, prior, and informed consent; ongoing during analysis of results as they emerge to limit the likelihood of surprise; and continuing during communication of findings with different stakeholders. Researchers also need to be aware of the potential for conflict from the beginning of CBA projects, and be careful not to overprivilege one source of knowledge or evidence if conflict does arise. In this way, CBA processes may help to create balance across different ways of knowing, helping to ensure that Western approaches do not repeat past mistakes where research undermined traditional knowledge and community perspectives.^38^, ^85^

- *Managing expectations*: the focus of CBA on building adaptive capacity and developing interventions can result in high expectations within partnering communities that projects will directly influence policy and programming. Yet, not all CBA projects have discernible outcomes, especially in the short term, and even where they do, may have difficulty in sustaining them or face significant barriers impeding success. Failure to manage expectations and the overselling of potential benefits by researchers can result in conflict with local partners if there is a mismatch in expectations. Indeed, funding agencies increasingly want to see decision‐making and action‐oriented outcomes as explicit objectives in project proposals, often supported by letters of support from communities, pushing researchers to sometimes list outcomes which may or may not happen and to which partners may or may not be committed. Managing research promises and funding agency demands is important for creating realistic expectations of CBA projects.
- *The multifaceted role of the researcher*: Researchers (be they students, faculty, consultants, community members, or government‐ or NGO‐based) play various roles in CBA projects, including as educators, communicators, community workers, promoters, facilitators, and negotiators. These roles reflect the nature of CBA work, where researchers are continuously asked and challenged by community partners to go beyond the standard academic practice of only generating new knowledge. Such roles attract many to CBA research; yet such demands and responsibilities can also compromise the integrity and quality of the research component of projects. For example, a number of workshop participants reported being frequently requested to provide educational resources and training outside of the research parameters, requested to search out funds for other projects, and asked to advise on other topics. While providing this level of support was deemed rewarding and critical for establishing trust, the time commitments required can have implications for the quality and impact of the actual research. It is important, therefore, for researchers to be open about these challenges, and negotiate relationships with community partners in a transparent manner, starting at the onset of a project.

### Responsibilities of the Researcher in Northern CBA

In pursuing CBA approaches, researchers need to scrutinize carefully the context in which they are working, and be cognizant of the challenges and benefits CBA brings. There is no ‘one size fits all’ guideline of how to do CBA effectively, but there are a number of responsibilities for those engaged in CBA work:

- *Researchers should not assume that research has a positive role to play in community adaptation just because it utilizes participatory approaches*. CBA can assist communities, build capacity, develop locally appropriate adaptations based on indigenous and Western knowledge, expand the salience of planning for future risks, and direct attention to locally important issues. Yet, participation in and of itself does not necessarily denote good or ethically sound CBA. In fact, participation in CBA research can perpetuate the privilege of Western knowledge over local values and indigenous knowledge, and can further marginalize communities if power relations are not addressed (also see Refs 28, 33, 84). CBA also does not prevent maladaptation (see Table 1), and in conducting research that facilitates or legitimizes intervention, researchers must address the potential that their work can do harm, especially when constrained by a lack information to make robust decisions in light of dynamic climate/ecological conditions. These potential dangers require careful consideration before projects proceed.
- *Researchers need to manage expectations and be realistic about what CBA can achieve*. While contending with the possibility that CBA can effect harmful change, we also must address the risk of ineffectuality. There are multiple barriers to adaptation that limit what can be achieved locally, and many of the purported benefits of CBA—e.g., capacity building and empowerment—may appear tokenistic to communities facing many pressing issues. Given the history of research in the North, deficits between what projects promise and what transpires can further erode trust in research. Being transparent with communities about expectations is thus essential for CBA work, and needs to begin with researchers and partners recognizing that CBA is not a ‘silver bullet,’ with work unlikely to overcome many of barriers to adaptation in the short term. Framing and communicating research‐funded/‐driven community adaptation initiatives as ‘pilot projects’ is one way to help moderate expectations; highlighting the importance of projects as first steps for increasing the political salience of adaptation and communicating local needs to higher levels is also important. Careful selection of methods, such as incorporating process‐based evaluation techniques in addition to outcomes‐oriented evaluation, may also help make legible the short‐term results or progress of CBA programs.
- *Researchers should premise their work on an understanding that in CBA*, *how knowledge is generated is as important as the research outcomes*. Researchers need to be cognizant of key principles of CBA, including codeveloping projects with communities who are engaged as partners, utilizing locally appropriate and approved theories and methodologies that respect the history, culture, values, and wishes of a community, while recognizing that each community is different in terms of their expectations and needs. Researchers working in indigenous communities also need to understand the colonial history and culture in which they work, and consider the underlying power dynamics shaping collaborations. Personal relationships and reciprocity underpin these principles,^72^, ^82^, ^115^ yet projects may fail or encounter substantial challenges even if these principles are adhered to; this is the very nature of CBA. Such challenges and failures should not be sanitized when work is presented but fully reported on. Accurately describing methodological processes and actions undertaken to secure community acceptance and support is essential for broader learning by the CBA community. Moreover, although ethical social science research always requires community consent, researchers should not necessarily be dissuaded from topics that do not imply or require a high degree of participation, or from initiating research ideas that may be of merit but fall outside immediate community priorities (such as maintaining a climate change focus, including future orientation).
- *Researchers must be flexible and open to change*, *acknowledging that community needs and perspectives may evolve as the project advances*. Flexibility implies the need for constant reflective attention, a consideration noted in decolonizing methodologies in general.^116^, ^117^ For example, theories and methods guiding projects considered participatory by researchers, may be viewed quite differently by communities as the work evolves, and may reinforce existing power differentials. Flexibility needs to also be balanced, however, within the context of the adaptation focus of CBA projects and skills of the researchers involved. Depending on the project and community interest, this may involve linking adaptation to more immediate socioeconomic concerns that also act as determinants of vulnerability to climate change (e.g., Ref 50), or more explicitly trying to bring in an adaptation lens.
- *Relationships and reciprocity are essential for building the trust in community‐based projects necessary for successful engagement*. The importance of developing long‐term relationships is essential for CBA, along with the need for regular interaction with communities and emphasis on the sharing and discussion of results. For students engaged in relatively short‐term projects, the ability to contribute to existing or ongoing projects through larger team‐based and collaborative projects is important for maintaining long‐term commitments, and to avoid short‐term ‘parachuting in’ of researchers. Relationship building does not necessarily require researchers to spend extended time in communities, although depending on the situation it can be helpful, and needs to be assessed on a case‐by‐case basis. Because a key component of CBA involves communities leading and partaking in the research process, continuous research presence could undermine this. The ability of researchers to ‘let go’ of successful projects and give space for communities to establish ownership over them is also important.
- *Researchers should work to better coordinate and plan CBA research in advance to avoid duplication and build on existing work*. There are often divergent perspectives between and among researchers and communities about the aims, objectives, and outcomes of CBA. Promoting transparency and accountability, supporting communities to help identify and characterize the potential benefits and tensions the work may bring, and flexibility and openness in design and approach are essential for projects to evolve positively and productively. The use of memorandums of understanding (MOUs) or partnership agreements have been advocated by some indigenous organizations and researchers in this context to minimize the potential for future conflict by formalizing expectations, decision‐making processes, and responsibilities among partners.^87^, ^118^ Equally, some communities view such formalized agreements as unnecessary or find formalization of arrangements inconsistent with local values for relational and reciprocal ethics.
- *CBA research requires advance planning and better coordination*. Another significant challenge faced by CBA researchers is that of effective coordination before and during projects, with communities often indicating that they are not ‘research‐fatigued’ but ‘researcher fatigued,’ pointing to a lack of coherence among research projects in the same location. As CBA research involves significant community engagement, duplication of the data collection and engagement processes across multiple projects can cause loss of interest and weariness among community members; equally, extensive engagement in developing projects that do not get funded can result in significant frustration and limit interest in future projects, especially given other pressing needs in communities. Addressing this requires specific communication and planning between researchers engaged in CBA‐work in the North. Researchers should actively seek‐out those working in communities they plan to also work in, noting the importance of on online databases of research activity that facilitate understanding of who is doing what (e.g., through websites such as that maintained by Nunavut Climate Change Centre), along with targeted literature reviews and gap analyses.^119^

In meeting these responsibilities, personal skills have an important role. Key traits necessary for work of this nature include deep listening, patience, openness to multiple ways of knowing, willingness to accept and respond to criticism, flexibility, self‐reflection, an ability to communicate and facilitate, a willingness to learn, a desire to develop and maintain strong and lasting relationships, and a sense of humor. These are not skills typically associated with an academic training or undergraduate/graduate university education. To manage these challenges in CBA projects, it is important that students are screened for suitability, are brought along for preliminary field visits to explore their capacity for fieldwork, are provided with sufficient pretraining in CBA and decolonizing research approaches, and are exposed to the challenges that may arise when conducting CBA research—all of which were identified by participants as essential to successful student training and development.

Similarly, for senior‐level CBA researchers, CBA projects demand a level of engagement, openness, shared decision making, uncertainty, and interdisciplinarity not typically associated with research in academia, with a variety of barriers to working on such projects well documented (e.g., recognition in tenure and promotion, time, incentives, etc.), see Refs 34, 72, 120. It has been argued that addressing such challenges requires a transformation in how scientific and academic institutions operate and students are trained, with increased emphasis on interdisciplinary and capacity building for coproducing knowledge with multiple users.^11^, ^121^, ^122^

## CONCLUSION

The last decade has witnessed the rapid emergence of CBA as a key component of adaptation research and practice, including in the Arctic. The growth in the importance of CBA in northern regions, however, has not been matched with critical reflections on what it means to do CBA, beyond statements on the importance of ‘engaging’ communities. To this end, this article covers novel ground, bringing together multidisciplinary academic researchers and emerging scholars to identify, examine, and reflect on the opportunities, tensions, and challenges of doing CBA with Inuit communities. We argue that although CBA is a powerful approach for supporting communities to adapt to climate change, researchers need to be aware of the challenges of such work and the potential maladaptive implications that may result. Indeed, we caution against the uncritical rush to adopt CBA approaches evident in much Arctic‐focused climate change research.

We also note the limitations of the article, emphasizing that the study only captures the perspectives of largely southern‐based, nonindigenous academic researchers. The issues profiled here are thus not definitive and are by no means exhaustive, but nevertheless represent the beginning of a larger conversation on CBA—one which needs to involve communities, local and regional governments, indigenous organizations, researchers, funders, and decision makers—deciding if, when, where, and how CBA has a role in emerging adaptation research. Indeed, this article can be viewed as part of the CBA process itself, involving colearning and continuous critical reflection to improve how we engage and interact with indigenous communities, knowledge systems, and cultural norms, epistemologies, and ontologies. The importance of such reflection is increasingly being recognized in an environmental change context in general and for adaptation in particular, and is essential for underpinning effective and ethical research.
